# Towards a Supertree of Arthropoda: A Species-Level Supertree of the Spiny, Slipper and Coral Lobsters (Decapoda: Achelata)

**DOI:** 10.1371/journal.pone.0140110

**Published:** 2015-10-13

**Authors:** Katie E. Davis, Thomas W. Hesketh, Cyrille Delmer, Matthew A. Wills

**Affiliations:** Department of Biology and Biochemistry, The University of Bath, Bath, United Kingdom; Sars International Centre for Marine Molecular Biology, NORWAY

## Abstract

While supertrees have been built for many vertebrate groups (notably birds, mammals and dinosaurs), invertebrates have attracted relatively little attention. The paucity of supertrees of arthropods is particularly surprising given their economic and ecological importance, as well as their overwhelming contribution to biodiversity. The absence of comprehensive archives of machine-readable source trees, coupled with the need for software implementing repeatable protocols for managing them, has undoubtedly impeded progress. Here we present a supertree of Achelata (spiny, slipper and coral lobsters) as a proof of concept, constructed using new supertree specific software (the Supertree Toolkit; STK) and following a published protocol. We also introduce a new resource for archiving and managing published source trees. Our supertree of Achelata is synthesised from morphological and molecular source trees, and represents the most complete species-level tree of the group to date. Our findings are consistent with recent taxonomic treatments, confirming the validity of just two families: Palinuridae and Scyllaridae; Synaxidae were resolved within Palinuridae. Monophyletic Silentes and Stridentes lineages are recovered within Palinuridae, and all sub-families within Scyllaridae are found to be monophyletic with the exception of Ibacinae. We demonstrate the feasibility of building larger supertrees of arthropods, with the ultimate objective of building a complete species-level phylogeny for the entire phylum using a divide and conquer strategy.

## Introduction

The Achelata are part of the highly diverse Decapoda, and are typically classified into five families; the extant Palinuridae (spiny lobsters), Scyllaridae (slipper lobsters) and Synaxidae (furry or coral lobsters), plus the extinct Cancrinidae and Tricarinidae. Despite their common names Achelata and Nephropidae (true lobsters) are not sister groups. Monophyly of Achelata is supported by all formal analyses, but the relationships and monophyly of the three constituent families remain equivocal. Morphological data support monophyly of all three extant families, while fossil data imply that Palinuridae are paraphyletic with respect to Synaxidae [1]. The most complete molecular phylogeny [1] concatenated data from five genes (18S, 28S, H3, 16S and COI) for 35 taxa, and found support for just two major clades; Scyllaridae and a group comprising Palinuridae and Synaxidae, with the latter placed within Palinuridae. Despite the generation of a number of smaller data sets and trees focussing on particular genera, the issue of deeper achelatan phylogeny has not been revisited; neither have more inclusive trees been inferred. Here, we synthesise the corpus of published trees using a supertree approach, specifically to investigate its efficacy and to test the suitability of our methods and new software [2] for application to further analyses.

Supertree methods offer a practicable means by which to synthesise large numbers of smaller trees with partially overlapping leaf sets. These “source trees” can themselves have been inferred from any type of data (e.g., morphology or molecules), each using the particular set of analytical approaches deemed most appropriate by their authors (e.g., distance methods, variously complex parsimony, likelihood or Bayesian frameworks). This inclusivity contrasts with supermatrix approaches containing diverse data types. In such cases, it is necessary either to apply a single analytical model across all characters [3–5], or to use the trees from the optimal analysis of one data type (e.g., the maximum likelihood analysis of molecular data) to construct a constraint tree or scaffold for the analysis of another (e.g., parsimony analysis of morphology) [6].

Large, inclusive and complete (i.e., all known species) phylogenies are vital for a variety of applications in evolutionary biology, ecology and conservation. Inter-nested patterns of shared evolutionary history mean that the traits and attributes of species are expected to share a complex, hierarchically correlated structure. Ecological or behavioural studies of trait correlations and interactions must therefore include phylogenetic information so that the conflated effects of shared ancestry can be factored out [7–12]. Similarly, the value of conserving a particular species may be at least partially determined by its “evolutionary distinctiveness”; the extent to which it is phylogenetically distant or distinct from its nearest relatives [13].

Supertrees have been produced for a diverse array of major clades, although there has been considerable emphasis upon vertebrates; notably dinosaurs [14,15], crocodiles [16], mammals [17] and birds [18]. Surprisingly, there have been few published arthropod supertrees, despite the successful application of the approach to Adephaga (Coleoptera) at the level of genera [19], and at the family level to Odonata [20] and Hymenoptera [21]. Arthropoda probably contains between 2.5 and 10 million species, although estimates as high as 30 million have been obtained [22–24]. Arthropods constitute more than 80% of described animal species, are abundant in virtually all habitats, and are of global economic [25] and ecological [26] importance. The ecosystem service value of arthropod pollinators alone is estimated at €153 billion per annum [25]. Given their pivotal ecological role in many habitats, a major loss of arthropod biodiversity would have catastrophic and far-reaching consequences [26]. At the same time, new species of arthropods (and especially insects) are being described at a rate exceeding that for all other animal, fungal and plant groups [27] with the conservation status of these species usually being unclear. An efficient means for synthesising the wealth of phylogenetic inferences for arthropods would therefore be valuable. We have chosen Achelata as an initial study clade since they are relatively small and well-documented, and contain representatives that have both consumptive (food source) and non-consumptive economic value (e.g., Palinuridae) [28].

## Methods

### Source Tree Collection

Potential source trees were identified from online resources. The Web of Knowledge Science Citation Index [29] was searched from 1980 to 2013 using the search terms: phylog*, taxonom*, systematic*, divers*, cryptic and clad* in conjunction with all scientific and common names for the Achelata from infra-order to sub-family level. All papers mentioning or implying the existence of a tree in their title or abstract were examined. In addition, the references cited by these papers were trawled for additional sources. All source trees and selected meta-data were digitised in their published form using TreeView [30] and the Supertree Toolkit (STK [2]). The latter is a fully integrated set of scripts designed to process trees and meta data, and to output matrices for MRP [31] supertree analysis or sets of trees for analysis using other supertree methods. The new version is either GUI or command line driven and offers much greater flexibility and functionality than its precursor that constituted a user defined processing pipeline [32]. Meta-data included bibliographic information, the types of characters used (e.g., molecular or morphological) and the methods used for tree inference. No corrections were made for synonyms or any other apparent errors or inconsistencies in the source trees prior to processing.

All the source tree data were deposited into our new resource at the Supertree Toolkit website [33]. This resource comprises a searchable, freely available database. All our source trees are archived here as they appear in their original published form, along with meta-data that allow further analyses re-purposing. This resource fills an important nîche, as few authors make their trees available in machine-readable form. All source trees curated for our arthropod supertrees will ultimately be archived here.

### Data Processing

The tree presented here differs from all other previously published supertrees as it utilised the STK in order to standardise and partially automate the process of construction. It is vital to ensure that source trees are treated in a consistent and repeatable manner in assembling a supertree [34,35]. The STK was devised in order to increase the accuracy and uniformity of approach, as well as to speed data processing. We followed the protocol described by Davis and Page [18].

Once data collection and data entry were complete, we ensured that source trees met several criteria before inclusion in the analysis:

1. Only trees presented by their authors explicitly as a reconstruction of evolutionary relationships were included. We therefore excluded taxonomies and informal phylogenies (i.e., we only included those derived from an explicit matrix of characters).
2. Only phylogenies comprising clearly identified species, genera or higher taxa and clearly identifiable characters were included.
3. Only trees derived from the analysis of a novel, independent dataset were included.

Non-independent studies were defined as those that utilised identical matrices (i.e., the same taxa and characters), or where one matrix was a subset of the other. In the former case, the “identical” source trees were weighted in inverse proportion to their number. In the latter case, the less inclusive tree was removed from the data set.

OTUs (operational taxonomic units) were standardised to reduce the inclusion of higher taxa, and to remove synonyms and vernacular names (which were standardised using the freely available online WoRMS database [36]). Where authors used higher taxa as proxies for particular exemplars, we substituted those with the names of those genera or species. Where no exemplars were specified, higher taxa were removed from source trees by substituting those constituent taxa present in other source trees as a polytomy in the focal tree. This avoided artificial inflation of the data set. Definitions for higher taxa were derived from WoRMS [36].

Taxonomic overlap was checked once the nomenclature had been standardised. Each source tree required at least two taxa in common with at least one other source tree [37]. Overlap within our dataset was sufficient; therefore no source trees were removed and we were able to proceed to matrix creation without any further edits. See S1 File [38] for the source trees as they were included in the analysis, S2 File [38] for a reference list of all source trees and S3 File [38] for the STK data file. Source trees in their original form were deposited in the Supertree Toolkit website database [33].

### Supertree construction

Achelata are a relatively small group (~150 species), and our data set contained 531 species (118 ingroup) from 55 source trees (S3 File [38]). Our supertree was inferred using Matrix Representation with Parsimony (MRP; [31]); the most commonly used and most tractable approach with medium to large data sets [39]. Source trees were encoded as a series of group inclusion characters using standard Baum and Ragan coding [31], and automated within the STK software. All taxa subtended by a given node in a source tree were scored as “1”, taxa not subtended from that node were scored as “0”, and taxa not present in that source tree were scored as “?”. Trees were rooted with a hypothetical, all-zero outgroup [40]. The resulting MRP matrix (S4 File) was analysed using standard parsimony algorithms in TNT [41]. We used the “xmult = 10” option, and ran 1000 replicates for the analysis, each using a different random starting point for the heuristic search. This improved exploratory coverage of the tree space, potentially avoiding local minima in the solutions.

## Results

**Fig 1:**
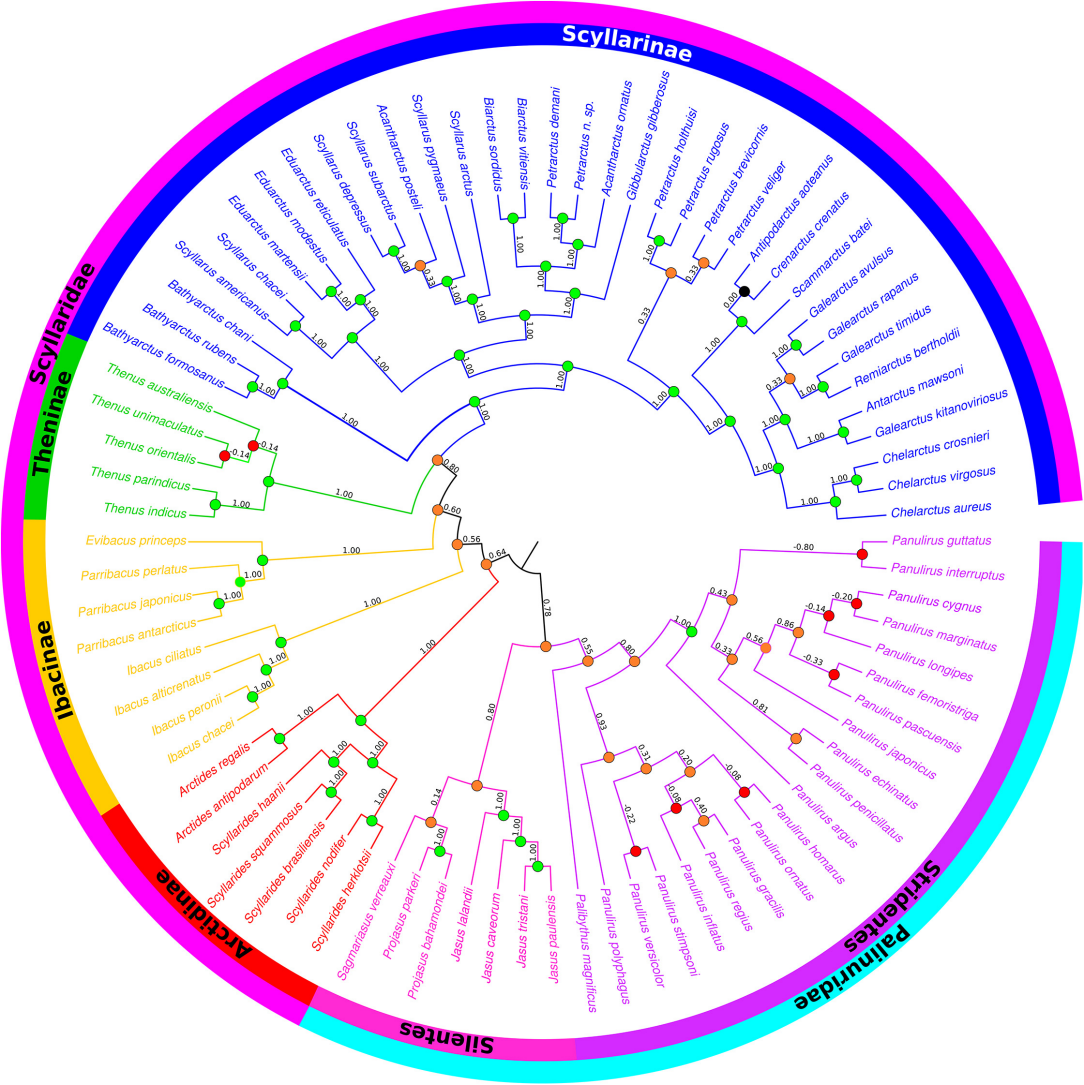
Maximum Agreement Subtree (MAST). The MAST was calculated for 3000 MPTs of length 2889 steps with V scores superimposed (V+ not shown). Green circles denote nodes with V scores of 1, orange denote nodes with V support between 0.01–0.9, black denotes V support of 0 and red denotes negative V support. The tree was generated using FigTree [43].

The analysis found 3000 MPTs of length 2889 steps. We then computed a Maximum Agreement Subtree (MAST) in PAUP* 4.0b10 [42]; the resulting tree comprised 82 ingroup taxa and was fully resolved. Fig 1 shows the complete tree, see S5 File [38] for the MAST in Newick format.

We calculated support for each bifurcating node in the supertree using the V index [44]. Each source tree was examined to determine if it contained a given supertree node. V indices range between +1 (where all source trees contain the node) and -1 (where no source trees contain it). Values over zero are consistent with support in the majority of source trees. A more relaxed index, V+, also takes permitted relationships (i.e., those consistent with polytomies in the source trees) into account, and therefore tends to yield higher values than V. All deep nodes in the supertree received positive V and V+ scores. Only nine nodes in the supertree received a negative V score, and just one had a negative V+. One additional node received a V score of zero. The negative values were all found towards the tips of the supertree and within the genera *Panulirus* and *Thenus*. This suggests that the fundamental splits in the tree are very well-supported and that only the more recent, species-level relationships show significant discordance in the source trees.

The supertree contains 56% of all described species of Achelata, and synthesises source trees published from 1992 to 2013 (no suitable trees were found from the period 1980–1991). Two clades were removed in the MAST consensus: *Justitia*/*Linuparus* and *Palinurus*. This suggests that these taxa are highly mobile within the source trees and could benefit from further study. Other taxa removed by the MAST were those that are poorly represented and/or poorly constrained within the source trees. For example, *Ibacus pubescens* is only present in one source tree.

Bininda-Emonds and Bryant [45] noted that the MRP method can lead to the creation of spurious clades and relationships that are not present in any of the source trees (“novel clades”). Although simulations have suggested that such anomalies are unlikely to be a significant problem [46], empirical studies have found an incidence of novel clades affecting up to 3% of taxa in the study [18]. However, no novel clades were found in this analysis.

**Fig 2:**
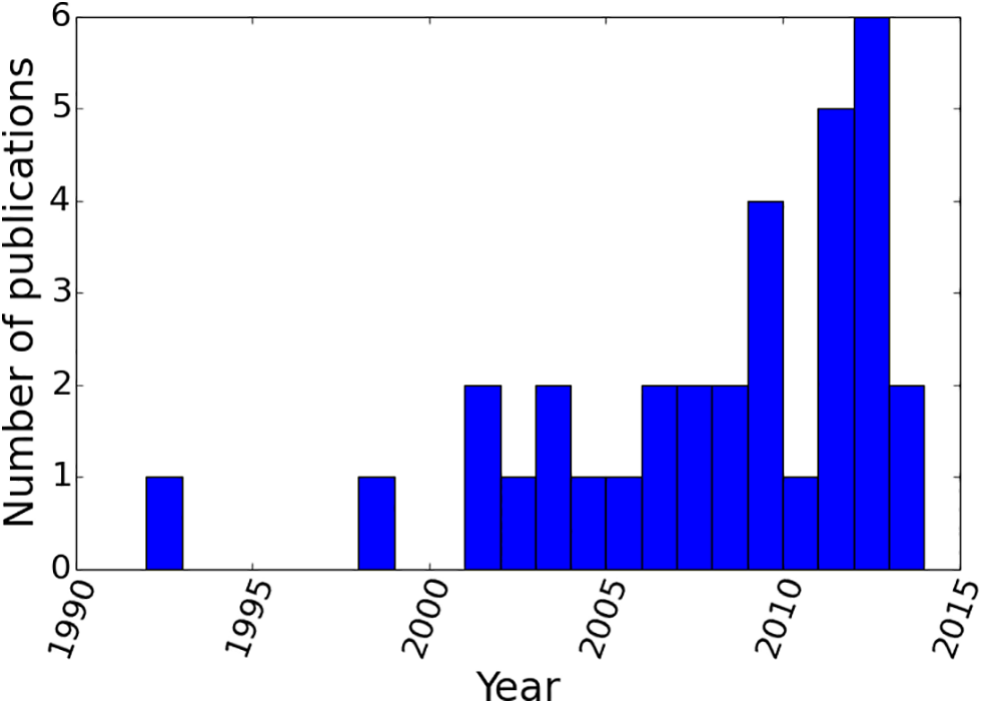
Number of phylogenies published by year. The number of phylogenies published, and included in the supertree analysis, is heavily skewed towards recent years with relatively few trees from pre-2000.

**Fig 3:**
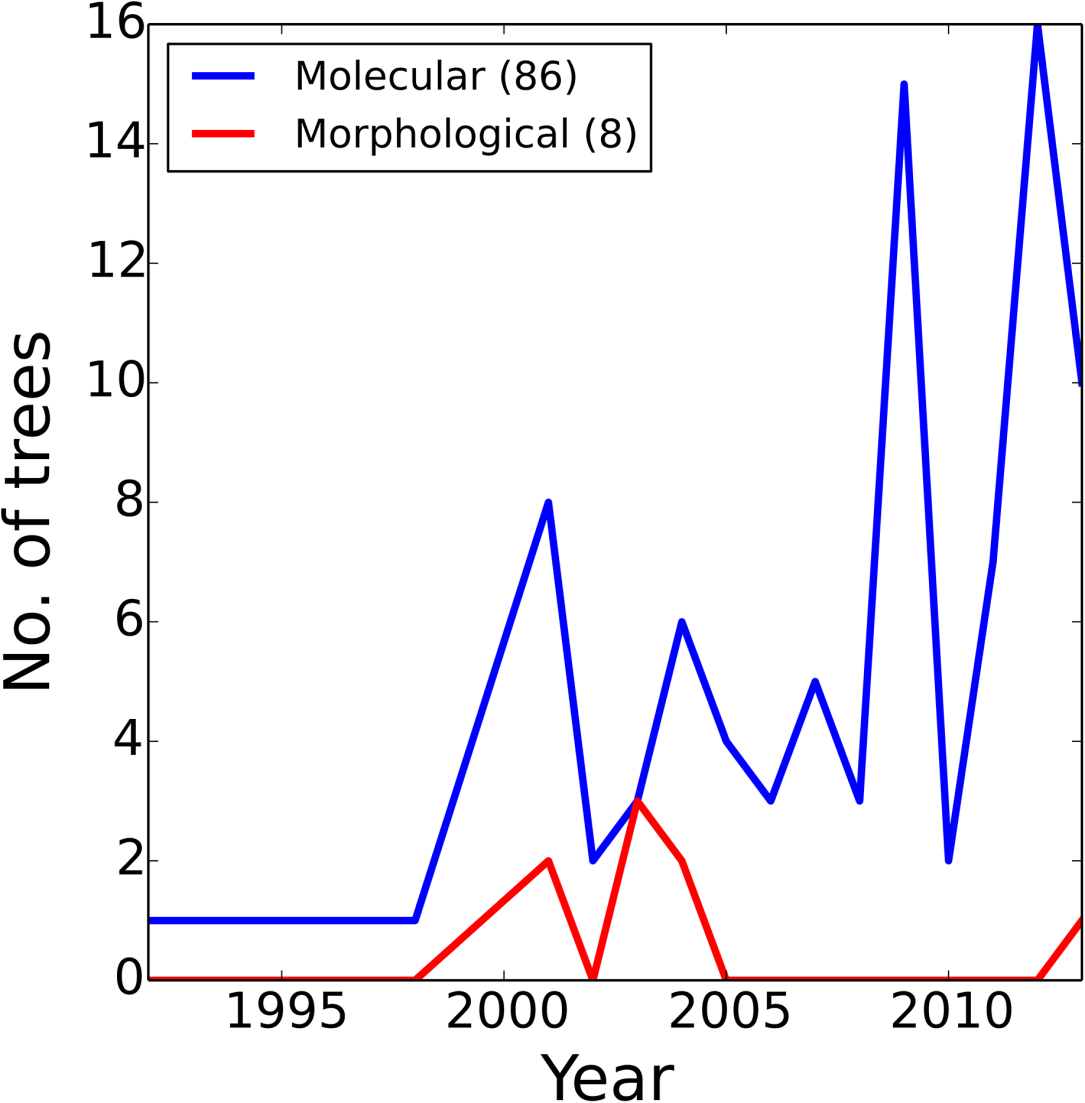
Source of character data by year. There is a strong bias towards source trees derived from molecular data for all years and increasingly so from 2000 onwards.

**Fig 4:**
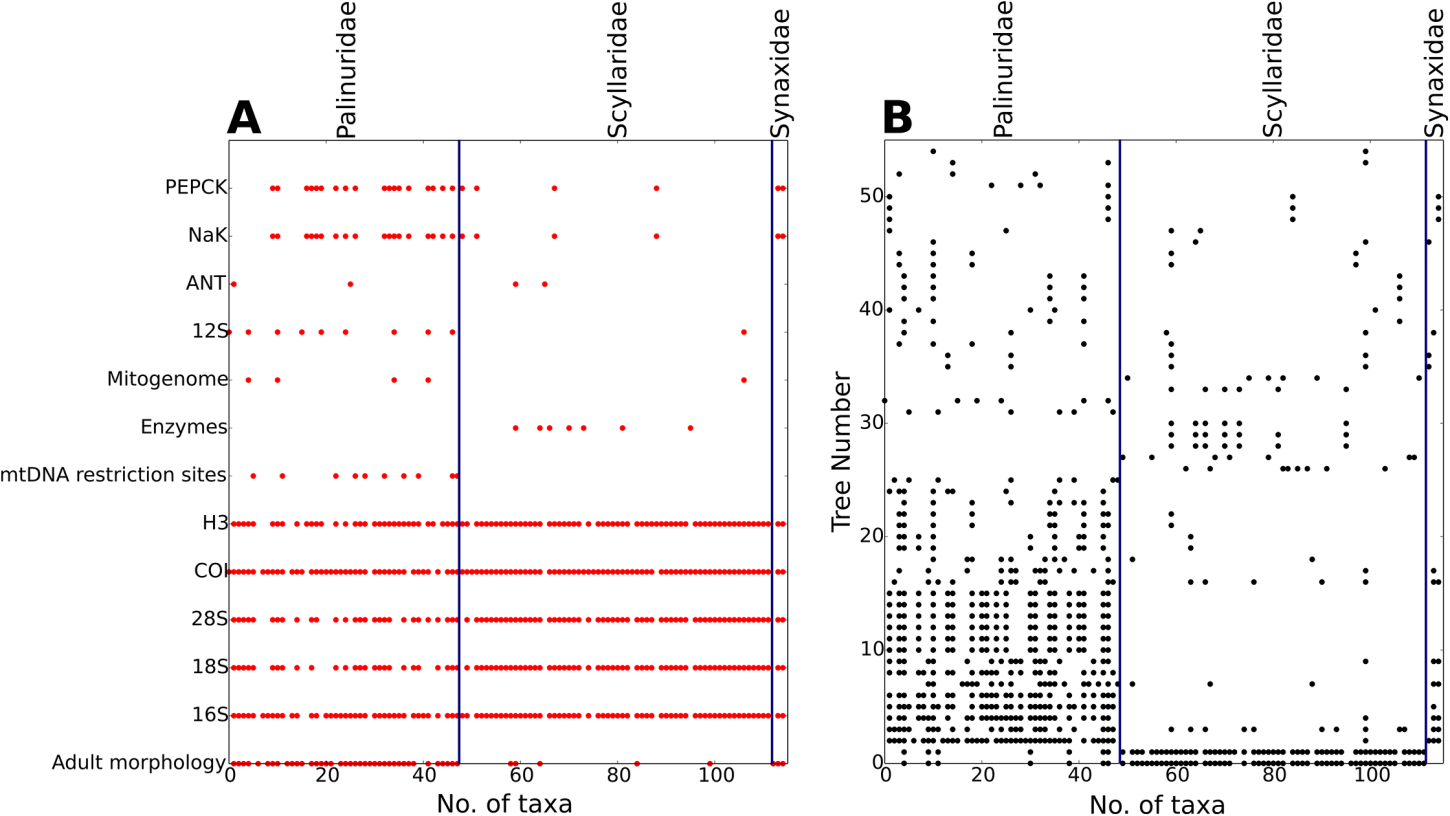
A) Characters sampled per taxon, partitioned by taxonomy. Palinuridae are better sampled than either Scyllaridae or Synaxidae for 50% of the characters used to build source trees. B) Number of taxa and presence/absence in source trees, partitioned by taxonomy. Palinuridae are better represented in the source trees than either Scyllaridae or Synaxidae.

**Fig 5:**
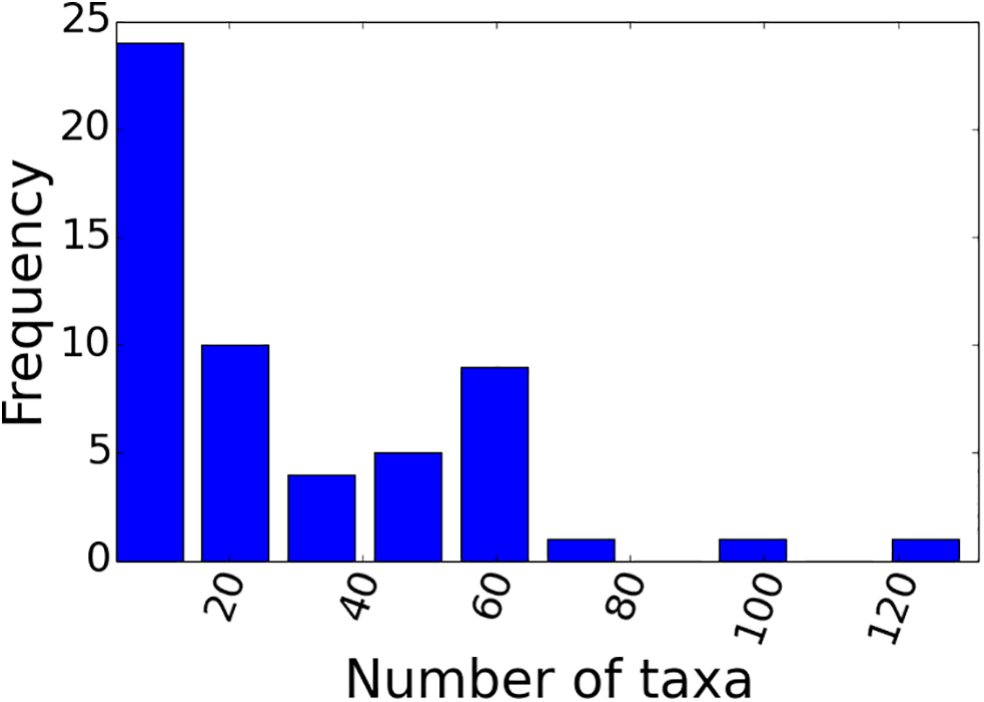
Source tree size shown by numbers of taxa present in source trees. The data set is dominated by source trees with only a small (<20) number of taxa.

The majority of our source trees were derived from papers published post-2000 onwards (Fig 2), and the majority were derived from molecular characters, reflecting the increasing abundance of molecular studies in the literature (Fig 3). Although some data types have broad taxonomic coverage (e.g., adult morphology and ribosomal RNA genes), many are absent for large numbers of taxa (Fig 4A). Some of these (e.g., enzymes and mitochondrial DNA restriction sites) have been used only rarely in recent years, but even some otherwise commonly-sequenced genes (e.g., 12S RNA and COI) have not been sequenced for large numbers of taxa. Plotting taxon presence/absence in source trees and partitioning by family (Fig 4B) reveals that the source trees show a strong taxonomic bias; far better data coverage for Palinuridae than for Scyllaridae or Synaxidae. We also note that the size distribution of our source trees was strongly skewed towards small source trees with fewer than 20 taxa (Fig 5).

## Discussion

### Achelatan Phylogeny

The Achelata supertree is fully-resolved and support is high throughout: 87% of nodes receive positive scores for both V and V+ indices (i.e., support or “permission” in the majority of the source trees for the V and V+ indices respectively). A well-supported, monophyletic Achelata is recovered, itself composed of large clades with high V indices; the Scyllaridae clade and the Palinuridae+Synaxidae clade. Synaxidae derive from within a paraphyletic Palinuridae, as reported by Palero *et al*. [1].

Within Palinuridae, Silentes and Stridentes are recovered as sister clades. The third proposed palinurid clade [1], comprising *Linuparus* and *Justitia*, does not have any taxa represented in the MAST phylogeny so cannot be assessed. Both Silentes and Stridentes receive high V and V+ scores (Silentes – 0.8; Stridentes – 0.55). All genera within Palinuridae are recovered as monophyletic with V and V+ scores of 1, with the exception of *Panulirus* which has a score of 0.8.

Within Scyllaridae all sub-families are recovered, with the exception of Ibacinae. The latter are split into two clades (*Thenus* and *Evibacus*/*Parribacus*) that are paraphyletic with respect to the Scyllarinae; a finding that is reflected in the source trees [1,47,48]. All sub-families and the two clades of Ibacinae have V indices of 1.00. All genera in the subfamilies Arctidinae, Ibacinae and Theninae are monophyletic, and also have V scores of 1.00. Within Scyllarinae all genera with the exception of *Acantharctus*, *Scyllarus*, *Petrarctus* and *Galearctus* are monophyletic, again all with postitive V and V+ scores. The polyphyly of *Acantharctus* is supported by the source data [48] clustering with *Petrarctus* and *Scyllarus*. The splitting of *Scyllarus* to cluster with *Eduarctus* and the inclusion of *Acantharctus posteli* are both relationships supported by the source trees [1,47,48]. *Petrarctus* is also split into two clades; again supported by the source trees [48,49]. The non-monophyly of *Galearctus* also reflects the source trees [49,50].

### Towards a Supertree of Arthropoda?

Our species-level supertree of Achelata was constructed using a protocol [18] implemented in newly updated and freely available software; the Supertree Toolkit (STK) [2]. The STK allows users to input trees and meta-data via a full GUI, and implements numerous functions including the standardisation of taxonomy, the substitution of higher taxa, checks for adequate overlap between source trees and the down-weighting of non-independent source trees (many of these outputting graphical summaries). The protocol and software allowed us to build this tree relatively quickly, and also archived data in a form (XML) that can be readily re-analysed and re-purposed by other workers. We will utilise this approach to generate further supertrees of Arthropoda: a phylum that has received surprisingly little attention from supertree workers.

Our supertree represents the source data well. Despite containing only 56% of described species, it is nevertheless the most complete phylogeny of Achelata produced to date. We highlight taxa that appear to be in need of further study as well as areas of the tree that are well-supported.

Supertrees are now widely accepted as a valid means of obtaining large, complete phylogenies relatively quickly, and without the need to collate and analyse primary data. As such, they maximise the value of source trees already in the literature. Each of these are constructed by experts on their focal groups, and usually represent the investment of considerable analytical time and computational resources. Although the size of supermatrices that can be analysed within tractable time frames is increasing, there is still disagreement and incomplete overlap between the results of phylogenomic and other large studies. Supertree methods are therefore likely to remain important as a means to synthesise trees resulting from these largest of analyses.

Supertrees have many potential applications in comparative biology and macroevolutionary studies; published examples include diversification rates through deep time, origins of modern taxa, and origins of species richness [14,20,21]. However they are particularly useful in the fields of comparative trait analysis and conservation, where large and inclusive cladograms are needed in order to remove the effects of phylogenetic correlation and to quantify evolutionary distinctiveness respectively [51,52]. Yet, their utility could be further enhanced in a number of ways.

1. Although supertree methods readily allow the inclusion of trees derived using any method from any type of data, there are still likely to be many described species that are not included in *any* primary phylogenies. The inclusivity of supertrees is therefore limited by the progress of the wider systematic community, although the largest trees are likely (almost by definition) to be supertrees at any given time. We will explore methods for utilising taxonomic information, thereby allowing supertrees to include all described species prior to their inclusion in published phylogenies. This will yield complete but unresolved supertrees that can be refined as new phylogenies are published.
2. At present, supertrees comprising hundreds or thousands of taxa can only be built within tractable search times using MRP methods. These parsimony supertrees contain no valid branch length information, and require time-consuming *post hoc* calibration (e.g., using fossils) in order to set them against any absolute or even relative time frame.
3. Although we found no rogue taxa in this study, the phenomenon of spurious clades in MRP supertrees is well-documented [45]. Bayesian and Maximum Likelihood supertree methods that may obviate such problems are being developed [53,54], but currently such approaches cannot handle data sets with more than a few tens of terminals.

Addressing these issues will enable the building of larger and more complete species trees without the problems associated with current supertree methods. Eventually supertree methods are likely to be rendered obsolete as computing power increases and more sequence data becomes available but they are still necessary for the foreseeable future; one way forward may be the combination of supertree approaches with supermatrix methods [55,56] as a means to “divide and conquer” large data sets.

## Supporting Information

S1 File Achelata source trees in their original published form. (TRE)

S2 File Bibliography for Achelata source trees. (BIB)

S3 File Supertree Toolkit file with Achelata trees and meta-data. (PHYML)

S4 File Achelata MRP matrix. (TNT)

S5 File Achelata MAST supertree. (TRE)
